# Compliance With Malaria Rapid Diagnostic Testing by Community Health Workers in 3 Malaria-Endemic Countries of Sub-Saharan Africa: An Observational Study

**DOI:** 10.1093/cid/ciw626

**Published:** 2016-12-06

**Authors:** Jan Singlovic, IkeOluwapo O. Ajayi, Jesca Nsungwa-Sabiiti, Mohamadou Siribié, Armande K. Sanou, Ayodele S. Jegede, Catherine O. Falade, Luc Sermé, Zakaria Gansane, Chinenye Afonne, Vanessa Kabarungi, Josephine Kyaligonza, Joëlle Castellani, Max Petzold, Melba Gomes

**Affiliations:** 1 UNICEF/UNDP/World Bank/WHO Special Programme for Research & Training in Tropical Diseases, World Health Organization, Geneva, Switzerland; 2 Department of Epidemiology and Medical Statistics, College of Medicine, University of Ibadan, Nigeria; 3 Child Health Division, Ministry of Health, Kampala, Uganda; 4 Groupe de Recherche Action en Santé, Ouagadougou, Burkina Faso; 5 Department of Sociology, Faculty of Social Sciences; 6 Department of Pharmacology and Therapeutics, College of Medicine; 7 Epidemiology and Biostatistics Research Unit, Institute of Advanced Medical Research and Training, College of Medicine, University of Ibadan, Nigeria; 8 Department of Health Services Research, School for Public Health and Primary Care, Maastricht University, The Netherlands; 9 Centre for Applied Biostatistics, Occupational and Environmental Medicine, Sahlgrenska Academy, University of Gothenburg, Sweden

**Keywords:** malaria, ACT, rapid diagnostic test, community health worker, compliance to test result

## Abstract

***Background.*** The World Health Organization recommends that all malaria management be based on parasitological identification. We monitored performance of trained community health workers (CHWs) in adhering to this recommendation to restrict artemisinin-based combination therapies (ACTs) to positive rapid diagnostic test (RDT)–confirmed cases in children in 3 malaria-endemic sub-Saharan African countries.

***Methods.*** In 33 villages in Burkina Faso, 45 villages in Nigeria, and 84 villages in Uganda, 265 CHWs were trained over a minimum of 3 days to diagnose malaria using RDTs (prepare, read, record results, and inform the patient about results) and treat RDT-confirmed uncomplicated malaria cases with ACTs. In Nigeria, CHWs were also taught to obtain a thick blood smear. Spent RDT kits and prepared blood slides were collected and interpreted independently in Burkina Faso and Nigeria to confirm CHWs' diagnoses. Interviews were held with 12 of 17 CHWs who prescribed ACTs for patients with RDT-negative test results, and with 16 of 29 caregivers to determine factors related to noncompliance.

***Results.*** Of 12 656 patients treated with ACTs in the participating countries (5365 in Burkina Faso, 1648 in Nigeria, and 5643 in Uganda), 29 patients (8 from Burkina Faso, 17 from Nigeria, 4 from Uganda) were RDT negative. The small number of RDT-negative ACT-treated cases limits statistical analysis. Only a few CHWs were involved, and they were more likely to be traders rather than farmers (odds ratio [OR], 6.15; 95% confidence interval [CI], 2.09–18.07; *P* = .0004). RDT-negative children who were treated with ACTs had a significantly higher probability of residing in a village other than that of the CHW (OR, 3.85; 95% CI, 1.59–9.30; *P* = .0018). Parental pressure was identified in interviews with parents.

***Conclusions.*** Noncompliance with results of RDT tests is relatively rare when CHWs are trained and well supervised.

***Clinical Trials Registration.*** ISRCTN13858170.

Despite improvements in control of malaria, the disease remains a leading cause of death in children in Africa [1]. Accurate diagnosis of malaria is part of case management. To target artemisinin-based combination therapy (ACT) to malaria-positive cases, the World Health Organization (WHO) recommends parasitological testing to confirm malaria before commencing treatment [2]. Prompt diagnosis and treatment is essential to prevent fatal malaria, to reduce the numbers of patients who can transmit malaria, and to rapidly identify patients with other causes of illness.

Malaria microscopy remains the reference method for malaria parasite diagnosis, but its value is often undermined because it needs equipment and skilled laboratory technologists and cannot therefore be done quickly, at the point of care. Antigen-based malaria rapid diagnostic tests (RDTs), introduced in the 1990s, have increased from a few products to >250 tests, which are submitted annually for evaluation and inclusion in procurement lists used by countries [3]. The availability of RDTs at increasingly competitive prices and their improved quality has substantially simplified and expanded diagnostic capacity in areas where microscopy was hitherto not practical. With substantial improvements in the number, quality, monitoring (product and lot testing), and reduced prices of RDTs available on the market, there has been an increased interest in scaling up approaches to improve malaria diagnosis using high-quality products. It is now possible to find RDTs used at peripheral facilities and shops and by trained community health workers (CHWs) to whom patients come for care. Use by trained CHWs residing in communities of patients widens access to diagnosis and accelerates treatment of malaria positive patients. Since 2013, all malaria-endemic countries have adopted WHO recommendations to test before ACT treatment. Diagnostic use (microscopy and RDTs combined) has now exceeded quantities of ACTs used for treatment in Africa [3].

Microscopy identifies parasites from a peripheral blood drop taken from a patient, whereas histidine-rich protein 2 (HRP2)–based RDTs detect antigen presence. All HRP2-based *Plasmodium falciparum* RDT strips use immunochromatographic methods to capture *P. falciparum*–specific antigens in lysed blood. A dye-labeled antibody binds to the parasite antigen and the resultant complex is captured on a nitrocellulose strip by a band of bound antibody, forming a visible “capture” or “test” line. The HRP2-based tests can pose problems as the antigens can remain in the blood for some time after successful treatment, confounding diagnosis when transmission is high and patients present with successive fevers within a short timeframe [4]. Neither microscopy nor RDTs establish malaria as the true cause of the illness, which complicates the differentiation of individuals who have only malaria vs those who have incidental parasitemia but another infection causing the illness. Studies have shown that persistent antigenemia detectable by HRP2-based RDTs occurs in 10% of patients [5]. False-negative results also occur and have more serious consequences because patients who have parasites are not detected and may be given inappropriate treatment; this phenomenon has been noted with HRP2 tests in the presence of low- and high-level parasitemia [6, 7] . Hence, the single most intractable problem with use of RDTs is trust in and compliance with RDT-negative results, particularly if the patient could have been treated to resolve the illness.

A recent systematic review conducted to evaluate health workers' compliance with RDT results indicates that compliance with positive and negative results is 97% and 78%, respectively [8]. CHWs in communities were more likely to comply with RDT-negative results (95%) compared with clinicians (75%) or clinicians and nurses (87%). Community use of RDTs [9–11] is not well documented, and the few studies carried out indicate that a much smaller fraction of patients (5.8%) are prescribed ACTs when RDTs are negative; all other studies monitoring health worker compliance with RDT results have observed health personnel in facilities [12–21]. We carried out a multicountry study to evaluate the effectiveness of a package comprising diagnosis of malaria with RDTs and treatment of positive cases with ACT by CHWs in 3 countries in sub-Saharan Africa [22]. Our aim in this substudy was to quantify the number of patients who were prescribed and treated with ACTs when the RDT was negative, and to understand the reasons and risk factors relating to this behavior.

## METHODS

In this study, an RDT was used to diagnose malaria on all patients coming for care, using whole blood collected via an aseptic finger prick [22]. Nationally approved RDTs were used: Malaria HRP2 (*Pf*) (Burkina Faso); CareStart (Nigeria); and Bioline SD (Uganda). In Burkina Faso, all RDT test strips were independently re-read by a supervisor. In Nigeria, thick malaria smears were also prepared from the same finger-prick blood sample, air dried, and stained with Giemsa stain and read/screened for the presence and quantification of malaria parasites under light microscopy at a magnification of ×1000 by an expert microscopist at the College of Medicine laboratory, University of Ibadan, Nigeria. Definitive parasite density was calculated assuming a white blood cell count of 8000 cells/µL [23].

Halfway through the intervention phase of the study (June 2015), we reviewed results of RDT-negative patients treated with ACTs in the 3 participating countries. We interviewed as many CHWs and guardians of the children treated with a negative RDT, to understand the circumstances leading to the practice. In Uganda, not all relevant CHWs or subjects could be successfully followed up. For CHWs available, we conducted structured interviews, which included precoded responses as well as an option for “open narratives.” In the structured questionnaire for the CHWs, we asked the CHWs 4 questions on (1) their normal patient assessment and treating procedures with an RDT-negative result; (2) reasons for prescribing ACTs for RDT-negative children; (3) perception of the risks related to treatment of an RDT-negative child; and (4) their behavior under parental pressure to treat a RDT-negative child.

We conducted key informant interviews (KIIs) with the guardians of children who were RDT negative and given ACTs to investigate their understanding of the RDT and its results, whether they were informed about the test result, and their understanding of what should happen with negative test results. In addition, their perception of performance of the CHW when their child was ill, the consequences of treatment with negative test results, and their motives for treatment regardless of test results were explored.

### Ethical Approval

Ethical approval was obtained from the WHO Ethics Review Committee and from national ethical review boards. Informed consent was obtained in accordance with the ethical standards set by the different ethics committees.

## RESULTS

Information on the demographic characteristics of RDT-positive and RDT-negative children treated with ACTs is provided in Table 1. Altogether, 12 454 febrile children were assessed, of whom 12 427 (99.8%) were RDT positive and 27 (0.2%) were RDT negative. Two afebrile children were treated. There was no difference in age or gender (the latter information was not collected in Uganda), but RDT-negative children who were treated with ACTs had a significantly higher probability of residing in another village than that of the CHW (odds ratio [OR], 3.85; 95% confidence interval [CI], 1.59–9.30; *P* = .0018).

**Table 1.** Artemisinin-Based Combination Therapy–Treated Patients With Positive and Negative Rapid Diagnostic Test Results, by Patient Characteristic

Characteristic	RDT Results, Read by CHW		Total
	Positive	Negative	
Total patients	12 627 (98.8)	29 (0.2)	12 656 (100)
Febrile patients			
Treated with ACT (Coartem)	12 427 (99.8)	27 (0.2)	12 454 (100)
Afebrile patients			
Treated with ACT (Coartem)	200 (99.0)	2 (1.0)	202 (100)
Sex^a^			
Male	3684 (52.7)	12 (48.0)	3696 (52.7)
Female	3304 (47.3)	13 (52.0)	3317 (47.3)
Not collected	5639	4	5643
Age			
≤11 mo	3018 (23.9)	10 (34.5)	3028 (23.9)
12–23 mo	2966 (23.5)	5 (17.2)	2971 (23.5)
24–35 mo	2799 (22.2)	3 (10.3)	2802 (22.1)
36–47 mo	2581 (20.4)	8 (27.6)	2589 (20.5)
48–59 mo	1232 (9.8)	3 (10.3)	1235 (9.8)
60–72 mo	9 (0.1)	0 (…)	9 (0.1)
Unknown	22 (0.2)	0 (…)	22 (0.2)
Village of the child^b^			
Child residing in same village as CHW	6413 (88.2)	14 (66.7)	6427 (88.1)
Child residing in different village from CHW	833 (11.5)	7 (33.3)	840 (11.5)
Unknown residence	24 (0.3)	0 (…)	24 (0.3)
Not collected	5357	8	5365

Data are presented as No. (%).

Abbreviations: ACT, artemisinin-based combination therapy; CHW, community health worker; RDT, rapid diagnostic test.

^a^ Not collected for Uganda.

^b^ Not collected for Burkina Faso.

Table 2 provides information that 8 of the 29 RDT-negative children treated with ACTs were from Burkina Faso, 17 from Nigeria, and 4 from Uganda. The overall error rate (ie, treatment of RDT-negative patients with ACTs) from CHW-determined RDT results were 0.2% (29/12 656): 0.1% (8/5365) in Burkina Faso, 1% (17/1648) in Nigeria, and 0.1% (4/5643) in Uganda. Table 2 shows the CHW-determined RDT results vs results of RDT strips re-read by supervisors in Burkina Faso and Nigeria. There is a clear bias toward interpreting the result as an RDT-positive test. In Burkina Faso, 96.6% of the RDT-positive results (read by CHWs) were verified as positive by the quality assurance (QA) procedure, but 3.4% of those read as positive by the CHW were determined to be RDT negative on QA; all the negative results were verified.

**Table 2.** Community Health Worker Test Results Versus Quality Assurance Test Results Versus Microscopy

Test Result	RDT Results, Read by CHW		Total
	Positive	Negative	
Total	12 627 (98.8)	29 (0.2)	12 656 (100)
QA RDT and microscopy results			
Burkina Faso			
CHW RDT results	5357 (99.9)	8 (0.1)	5365 (100)
RDT-positive QA result	5075 (96.6)	0 (…)	5075 (96.5)
RDT-negative QA result	177 (3.4)	5 (100)	182 (3.5)
RDT QA not available	105	3	108
Nigeria			
CHW RDT results	1631 (99.0)	17 (1.0)	1648 (100)
RDT-positive QA result	1207 (86.2)	3 (20)	1210 (85.5)
RDT-negative QA result	193 (13.8)	12 (80)	205 (14.5)
RDT QA not available	231	2	233
Microscopy positive	780 (65.8)	2 (15.4)	782 (65.2)
Microscopy negative	406 (34.2)	11 (84.6)	417 (34.8)
Microscopy unavailable	445	4	449
Uganda			
CHW RDT results	5639 (99.9)	4 (0.1)	5643 (100)

Data are presented as No. (%).

Abbreviations: CHW, community health worker; QA, quality assurance; RDT, rapid diagnostic test.

In Nigeria, a blood spot from the same finger-prick was used for RDTs plus a blood slide; microscopy was attempted for all patients, but in 449 cases the slides could not be read. Overall, 86.2% of the CHW-determined RDT-positive results were confirmed as positive on QA, but 13.8% (n = 193) of those read as positive by the CHW were assessed as RDT negative on QA. Among the 17 CHWs’ RDT-negative results treated with ACTs (with available QA), 80% were confirmed negative on RDT-QA. Of the 12 CHW RDT-negative results that were confirmed by QA as an RDT-positive result, 2 were smear positive, 8 were smear negative, and for 2 patients a smear result was not available. Of the 3 results read by the CHW as RDT negative where the RDT QA result was positive, all 3 were microscopy negative. Where microscopy results were available, 65.8% (780/1186) RDT-positive results were confirmed, but 34.2% (406/1186) of CHW-read RDT-positive results treated with ACTs were negative on microscopy QA. Although 84.6% (11/13) of RDT-negative results were confirmed through microscopy, 15.4% (2/13) were positive on microscopy, thus false RDT negatives. Uganda did not implement a QA procedure.

Table 3 provides information on the time of day at which the patient reached the CHW for treatment and the interval between onset of symptoms and arrival at the CHW for consultation, and provides information on the symptoms of the episode by RDT test results. The small number of RDT-negative cases limits statistical analysis. Generally, there was no difference between groups in factors related to delays in treatment or in baseline symptoms. Within the small number of RDT-negative children treated, there were higher proportions of children who presented with a history of repeated vomiting compared with RDT-positive cases (19.0% vs 9.2%), respiratory problems (19.0% vs 10.9%), and diarrhea (14.3% vs 9.9%). In contrast, RDT-positive ACT-treated children had a higher proportion of cases with fast breathing (10% vs 3.4%) and danger signs (0.1% vs none).

**Table 3.** Characteristics of Cases Prescribed Artemisinin-Based Combination Therapy, by Rapid Diagnostic Test Result

Characteristic	RDT Results, Read by CHW		
	Positive	Negative	Total
Total No. of children treated with ACT	12 627 (98.8)	29 (0.2)	12 656 (100)
Time of CHW consultation (24-h clock)^a^			
No.	6967	25	
Unknown	21	0	
Mean	11.8	14.5	
SD	4.6	4.6	
Not collected	5639	4	
Time of arrival at CHW^a^			
6 am–12 pm (morning)	4143 (59.3)	8 (32.0)	4153 (59.2)
12 pm–4 pm (afternoon)	1076 (15.4)	7 (28.0)	1083 (15.4)
4 pm–7 pm (evening)	1431 (20.5)	9 (36.0)	1440 (20.5)
7 pm–6 am (night)	316 (4.5)	1 (4.0)	317 (4.5)
Unknown	22 (0.3)	0 (…)	22 (0.3)
Not collected	5639	4	
Treatment delay: time between symptom onset and treatment, h^b^			
No.	1440	16	1456
Mean	28.6	23.6	28.6
SD	26.7	31.6	26.7
Unknown	191	1	192
Not documented	10 996	12	11 008
Delay in treatment after onset of symptoms (within 24 h)^c^	7270	21	7291
Treated within 24 h	5094 (70.1)	13 (61.9)	5107 (70.0)
Treated after 24 h	2082 (28.6)	7 (53.8)	2089 (40.9)
Unknown	94 (1.3)	1 (14.3)	95 (4.5)
Not documented	5357		
Symptoms on presentation			
Burkina Faso	5357	8	5365
Fever	5179 (96.7)	7 (87.5)	5186 (96.7)
Fast breathing	556 (10.4)	1 (12.5)	557 (10.4)
Danger signs	6 (0.1)	0 (…)	6 (0.1)
Ability to eat/suck	0 (…)	0 (…)	0 (…)
Nigeria and Uganda	7170	21	7291
Fever	7248 (99.7)	20 (95.2)	7268 (99.7)
Vomiting	671 (9.2)	4 (19.0)	675 (9.3)
Cough/difficulty breathing	789 (10.9)	4 (19.0)	793 (10.9)
Fast breathing^d^	710 (9.8)	0 (…)	710 (9.7)
Diarrhea	721 (9.9)	3 (14.3)	724 (9.9)
Danger signs	2 (<0.1)	0 (…)	2 (<0.1)
Ability to eat/suck	2 (<0.1)	0 (…)	2 (<0.1)
Other^e^	224 (3.1)	3 (14.3)	227 (3.1)
Referral for further management			
Child treated and referred	109 (0.9)	1 (3.4)	110 (0.9)
Child treated but no need for referral	12 484 (98.9)	28 (96.6)	12 512 (98.9)
Unknown	34 (0.3)	0 (…)	34 (0.3)

Data are presented as No. (%) unless otherwise indicated.

Abbreviations: ACT, artemisinin-based combination therapy; CHW, community health worker; RDT, rapid diagnostic test; SD, standard deviation.

^a^ Not collected for Uganda.

^b^ Only collected in Nigeria.

^c^ Not collected for Burkina Faso.

^d^ For Nigeria and Uganda, respiratory rate was only collected for children with elevated respiration.

^e^ Example of others by frequency: catarrh (n = 78); cannot play (n = 61); weakness (n = 53); headache (n = 51); chest indrawing (n = 10); stomach upset (n = 10).

Table 4 shows the CHW characteristics in relation to the occurrence of error in prescribing ACTs to RDT-negative children. Gender of the CHW has no influence, but there was a 5-fold higher risk of receiving ACT treatment after having an RDT-negative result when the CHW was single vs married, and the occupation of CHWs is important: Traders were 6 times more likely to prescribe ACTs to RDT-negative children than farmers, a conclusion confirmed by multiple regression analysis.

**Table 4.** Community Health Worker Characteristics, by Occurrence of Error in Prescribing Artemisinin-based Combination Therapies to Rapid Diagnostic Test Negative Patients

Characteristics	CHWs Who Never Gave ACTs to RDT-Negative Patients	CHWs Who Gave ACTs to RDT-Negative Patients	Total	Odds Ratio (95% CI); *P* Value
Total number of active CHWs	232 (93.2)	17 (6.8)	249 (100)	
Gender				
Male	78 (33.6)	5 (29.4)	83 (33.3)	
Female	153 (65.9)	12 (70.6)	165 (66.3)	1.22 (.43–3.45); .714
Unknown	1 (0.4)	0 (0)	1 (0.4)	
Marital status				
Married	199 (85.8)	11 (64.7)	210 (84.3)	
Single^a^	17 (7.3)	5 (29.4)	22 (8.8)	5.32 (1.73–16.5); .002
Unknown	16 (6.9)	1 (5.9)	17 (6.8)	
Education				
Primary	83 (35.8)	8 (47.1)	91 (36.6)	
Higher	118 (50.9)	9 (52.9)	127 (51.0)	0.79 (.30–2.07); .644
Unknown	31 (13.4)	0 (0)	31 (12.5)	
Occupation				
Farmer	166 (71.6)	6 (35.3)	172 (69.1)	
Trader	36 (15.5)	8 (47.1)	44 (17.7)	6.15 (2.09–18.07); .0004^b^
Other	26 (11.2)	3 (17.6)	29 (11.6)	
Unknown	4 (1.7)	0 (0)	4 (1.6)	
Years of experience as CHW				
None	4 (1.7)	0 (0)	4 (1.6)	
1–3	95 (41.0)	8 (47.1)	103 (41.4)	
>3	130 (56.0)	9 (52.9)	139 (55.8)	0.82 (.31–2.21); .876^c^
Unknown	3 (1.3)	0 (0)	3 (1.2)	

Abbreviations: ACT, artemisinin-based combination therapy; CHW, community health worker; CI, confidence interval; RDT, rapid diagnostic test.

^a^ Divorced and widowed included.

^b^ Trader vs farmer.

^c^ Over 3 years of experience vs 1–3 years of experience, tested for trend.

In Supplementary Table 1, “compliant” CHWs who did not treat an RDT-negative case with ACTs are compared with “noncompliant” CHWs for each country. There were a total of 249 CHWs who treated children with ACTs in the participating countries (49 in Burkina Faso, 42 in Nigeria, and 158 in Uganda), and a very small proportion of all CHWs (6.8% [17/249]) prescribed ACTs to RDT-negative patients. Separating CHWs who did not treat an RDT-negative case from those who did, noncompliant CHWs had substantial enrollment rates, and therefore experience (Supplementary Table 1). RDT-compliant CHWs had an average of 49.9 treated cases per CHW compared with an average of 64.6 treated cases for the noncompliant CHWs; the average is higher for every participating country as well as overall (108 vs 127 for Burkina Faso, 36.9 vs 45.7 for Nigeria, and 35.7 vs 43.5 for Uganda). The total number of erroneous treatments per CHW is low (minimum 1, maximum 3) reflecting the low overall error rate. The mean overall error rate for the 17 noncompliant CHWs was 2.6% (1.6% for Burkina Faso, 3.4% for Nigeria, and 4.5% for Uganda).

To check whether “noncompliance” occurred in new CHW recruits and immediately after training, when CHWs had less experience and might yield to parental pressure, we calculated the time from their first treatment to their first error. The mean number of days to their first error was 51.9 overall, 47.8 for Burkina Faso (minimum 0, maximum 112; SD, 55.8), 60.3 for Nigeria (minimum 0, maximum 174; SD, 59.4), and 14.5 for Uganda (minimum 10, maximum 19; SD, 5.2). Consequently, we cannot confirm a hypothesis that errors are higher in new CHW recruits (Supplementary Table 1).

### Qualitative Data

We successfully followed 12 of 17 CHWs providing ACTs to RDT-negative children—all 4 in Burkina Faso and 8 in Nigeria; we could not interview CHWs in Uganda. The Burkina Faso CHWs consistently indicated that they would not treat an RDT-negative child as this would be an error, but 2 CHWs responded that there are no consequences in treating a negative child; 1 of the remaining 2 CHWs was concerned about possible side effects and the other about developing resistance to malaria and progress of other untreated infections.

Nigerian CHWs had heterogeneous responses: More than half of the Nigerian CHWs confirmed treatment because guardians insisted. All responses (multiple responses were allowed) indicated they would use another drug to treat the child instead of not treating, and 6 of 7 CHWs said that they would refer the child to nearest health facility. They also indicated (5/8) that they would use ACTs for their own children if the child was RDT negative, as they had been using ACTs without RDTs in the recent past:

My belief is that the child will get well … children between the age of 4 months to 10 years have been using Coartem in the past, even before we started carrying out blood tests for malaria, and this drug has proven to be effective on these children. I have been familiar with its use and effectiveness for a long time. This is the reason why I could give Coartem to the child … the paracetamol I give them had finished and it was already late in the night so I cannot tell them to go and buy paracetamol from the next community. … The parent of the child said since I had no paracetamol, I was supposed to give the child Coartem. And they know Coartem is very effective. — CHW at Agunsa village, Nigeria, during KII

Only 1 CHW responded that the appropriate action was to treat the child with paracetamol (acetaminophen) or refer him to the health facility:

What I do is to give paracetamol and if there is no improvement I will refer to health centre … because they have better knowledge. Not only malaria presents with fever but people believe when a child is feverish, it is malaria. — CHW at Kupalo village, Nigeria, during KII

A few (3) mothers/parents of the children were interviewed in Uganda, 9 in Nigeria, and 4 in Burkina Faso. The results confirmed the CHW's statement and there was no sense of guilt, especially if the child improved:

… My child was weak and looked very sick, I explained to the [CHW], and he told me that it is malaria, and told me that he is going to give her medicine for malaria … and indeed after 2 days there was a change, but I know if the child is given medicine that does not treat the disease the child can become worse and can be taken on a drip in the facility … My child got better … as long as my child gets better, the [CHW] healed my child, the [CHW] did good. — Mother, Busaana village, Uganda, during KII

*When I took the child to the* [*CHW*]*, she asked me if the child was playing, and eating; She got something that is like a watch and counted the breathing rates, then after she explained to me why she needed to prick the child and take blood then test it for malaria. We waited for some time and she told me my child did not have malaria but according to me it seemed like he did because he was not eating and his body heat was high. I requested for the medicine though she was hesitant to give it to me … I requested and pleaded with her to give me the medicine because I felt the child was sick and maybe the testing had a problem and could not detect malaria and after some days under medication, he became fine that means he actually had malaria. — Mother, Migamba village, Uganda, during KII*

## DISCUSSION

In approximately 16 000 patients diagnosed with an RDT by trained CHWs in their malaria endemic areas [22], 12 627 RDT-positive patients and 29 of 2804 (1.03%) RDT-negative patients were treated with an ACT. These results from a very large community-based study confirm that CHWs are very reliable in using and complying with RDT results in targeting ACTs to patients with uncomplicated malaria. Withholding ACTs from febrile cases is of special concern, especially if the RDT readings are falsely negative; 2 of the 27 febrile RDT-negative cases were microscopy positive. Quantitative and qualitative analyses indicated that these ACT-treated RDT-negative cases were not errors made posttraining; they were made by experienced CHWs. Qualitative interviews with CHWs and parents confirmed that parental pressure and prior experience managing malaria with ACTs without RDTs played a role.

Only 3 prior studies carried out between 2006 and 2010 are known to have monitored community treatment and compliance with RDT results involving a total of 7328 patients assessed by trained CHWs in their communities in Uganda, Ghana, and Burkina Faso [10, 11, 24]. Microscopy was used to confirm RDT test results, and 5.8% of RDT-negative patients were prescribed ACTs. Factors affecting compliance have been identified as the malaria season, persistence of symptoms, lack of trust in RDT results, and patient pressure [11, 12, 15, 17, 20]; we found parental pressure and location/occupation of the CHW to be important. A recent meta-analysis indicates that CHWs comply best [8]. Our large study confirms this finding.

Until recently, the policy for treating malaria had assumed that most febrile illnesses were malaria, and that the risk of not treating with an effective antimalarial was greater than missing a true case. This approach probably saved many lives, but led to overuse of antimalarial drugs. When the cost of antimalarial drugs was lower than the cost of ACTs, microscopy was the primary method of diagnosis (not always of good quality, not often maintained, and often delaying treatment); antimalarial overprescription was tolerated, despite preventing other causes of fatal illnesses from being diagnosed and treated. With highly effective artemisinin drugs being the key component of ACTs and essential for treatment for both uncomplicated *and* severe malaria, *and* more expensive, there is increased focus on targeting treatment to malaria-positive cases through the use of more expensive RDTs, which become cost-effective when treatment is based on the result [24]. Multiple rounds of transparent evaluations now identify RDTs that consistently detect malaria at low parasite densities, and guide their purchase [3]. Introducing them into routine care, free of charge using trained CHWs, guided antimalarial treatment in remote communities. There was parental pressure, but CHWs bowed to such pressure very rarely.

There were some limitations to this study. We found both false-negative and false-positive results in patients on re-reading RDT test cassettes and comparing them with microscopy, supporting a legitimate concern that some children should have been treated in the face of negative RDT cassette results. The levels of overdiagnosis by CHWs who had received training were very small, but the constant presence of research staff in the communities, even where the study was being conducted by the Ministry of Health in Uganda, may have altered practice and compliance with RDT results.

## Supplementary Data

Supplementary materials are available at http://cid.oxfordjournals.org. Consisting of data provided by the author to benefit the reader, the posted materials are not copyedited and are the sole responsibility of the author, so questions or comments should be addressed to the author.

Supplementary Data
