# Supplementary material for: Compliance With Malaria Rapid Diagnostic Testing by Community Health Workers in 3 Malaria-Endemic Countries of Sub-Saharan Africa: An Observational Study
Source: Clin Infect Dis. 2016 Dec 6;63(Suppl 5):S276–82. doi: 10.1093/cid/ciw626 (PMC5146698; doi:10.1093/cid/ciw626)
Supplement: Supplementary Data [file supp_63_suppl-5_S276__index.html]

Supplementary Data 

# Compliance With Malaria Rapid Diagnostic Testing by Community Health Workers in 3 Malaria-Endemic Countries of Sub-Saharan Africa: An Observational Study

## Supplementary Data

Supplementary Data

- Supplementary Data - Pdf file
