# Supplementary material for: Compliance With Malaria Rapid Diagnostic Testing by Community Health Workers in 3 Malaria-Endemic Countries of Sub-Saharan Africa: An Observational Study
Source: Clin Infect Dis. 2016 Dec 6;63(Suppl 5):S276–82. doi: 10.1093/cid/ciw626 (PMC5146698; doi:10.1093/cid/ciw626)
Supplement: Supplementary Data [file supp_ciw626_ciw626supp.pdf]

Supplementary table 1: Performance of CHWs who prescribed ACTs by treatment experience, by site.

	Number CHWs	Total ACTs to RDT+ve cases	Total ACTs to RDT-ve cases	Mean days to first error	Error rate	Mean ACTs per CHW	SD	Min	Max	IQR - 25%	IQR - 75%
**All CHWs**											
Burkina	49	5357	8	-	0.1	109.5	64.6	19	343	64	141
Nigeria	42	1631	17	-	1.0	39.2	49	2	257	9	46
Uganda	158	5639	4	-	0.1	35.8	31.5	1	150	12	56
Overall	249	12627	29	-	0.2	50.9	51.8	1	343	15	69
**CHWs who gave ACTs to RDT negative patients**											
Burkina	4	508	8	47.8	1.6	127	47.9	94	197	96	158
Nigeria	11	503	17	60.3	3.4	45.7	55.6	2	206	23	43
Uganda	2	88	4	14.5	4.5	43.5	31.8	22	61	21	66
Overall	17	1099	29	51.9	2.6	64.6	60.8	2	206	23	94
**CHWs who never gave ACTs to RDT negative patients**											
Burkina	45	4849	-	-	0	108	66.1	19	343	64	141
Nigeria	31	1128	-	-	0	36.9	47.2	3	257	9	55
Uganda	156	5551	-	-	0	35.7	31.6	1	150	12	56
Overall	232	11528	-	-	0	49.9	51.1	1	343	14	68
